# Determinants of medication adherence in hemodialysis patients: a cross-sectional study based on capability-opportunity-motivation and behavior model

**DOI:** 10.1186/s12882-023-03231-0

**Published:** 2023-06-14

**Authors:** Mehdi Mirzaei-Alavijeh, Behrooz Hamzeh, Hamidreza Omrani, Sharareh Esmailli, Saeid Khakzad, Farzad Jalilian

**Affiliations:** 1 Social Development and Health Promotion Research Center, Health Institute, Kermanshah University of Medical Sciences, Kermanshah, Iran; 2 Research Center for Environmental Determinants of Health, Health Institute, Kermanshah University of Medical Sciences, Kermanshah, Iran; 3 Department of Internal Medicine, School of Medicine, Kermanshah University of Medical Sciences, Kermanshah, Iran; 4 Department of Health Education and Promotion, School of Health, Kermanshah University of Medical Sciences, Kermanshah, Iran

**Keywords:** Hemodialysis, Kidney patients, Medication adherence, Motivation

## Abstract

**Background:**

Medication adherence is a key component of successful dialysis in end-stage renal disease (ESRD). The aim of this study was to use the Capability-Opportunity-Motivation and Behavior (COM-B) model in order to identify the most important determinants of medication adherence among ESRD patients.

**Methods:**

This research was a cross-sectional design that was conducted in two steps in 2021. In the first step, COM-B components of patients undergoing hemodialysis (HD) therapy were extracted through literature review. The second step was a cross-sectional study among 260 ESRD patients referred to the dialysis unit from Kermanshah, in the west of Iran. Data was collected using a written questionnaire by interviews. The data was analyzed in SPSS version 16 software.

**Results:**

The mean age of respondents was 50.52 years [95% CI: 48.71, 52.33], ranged from 20 to 75 years. The mean score of medication adherence was 11.95 [95% CI: 11.64, 12.26], ranged from 4 to 20. Medication adherence is higher among patients with higher education (P = 0.009) and those who were employed (P < 0.001) and was significantly related to income (r = 0.176), while it was inversely and significantly related to the medication duration (r=-0.250). Motivation (Beta: 0.373), self-efficacy (Beta: 0.244), and knowledge (Beta: 0.116) are stronger determinants of medication adherence.

**Conclusion:**

COM-B model can be proposed as an integrated framework in predicting medication adherence among ESRD patients. Our findings provide theory-based recommendations that can help future clinical and research decision-making for the development, implementation, and evaluation of treatment adherence interventions in Iranian ESRD patients. The use of COM-B model can provide a comprehensive explanation about medication adherence in ESRD patients. Future research should be focus on increasing motivation, self-efficacy and knowledge of Iranian ESRD patients in order to increasing medication adherence.

## Introduction

Chronic Kidney Disease (CKD) is one of the main challenges in health systems that cause huge costs in healthcare for societies and governments [1]. CKD is a public health risk factor worldwide [2] that occurs during the progressive process of decreased nephron function [3]. In Iran, the prevalence and incidence rate of End Stage Renal Disease (ESRD) are about 357 and 57 per million each year, respectively, and statistics indicated a significant growth in the number of CKD patients in Iran, because the number these patients increase by 15% every year [4]. Currently, hemodialysis (HD) is the most common treatment method for ESRD patients [5, 6]. Based on the statistics reported by the Iranian Ministry of Health and Medical Education in 2016 the ESRD prevalence incidence has 30,284 patients and it is estimated annually that more than 4000 cases are added to the ESRD [7]. Several researches among dialysis patients show that they are prescribed a mean of 11–12 medications per day and these patients take, usually, 17–25 doses daily [8]. Thus, medication adherence is a key component of effective management of dialysis patients [9] that plays an important role in reducing the economic burden of the disease for families and society [10]. According to the World Health Organization (WHO), treatment adherence can be defined as the extent to which the patient’s behavior correctly, based on the instructions of the health care providers [11]. Treatment non-adherence is a major issue in chronic patients, including HD patients [12]. The average prevalence of treatment non‐adherence is 52.5% among HD patients [13]. Various determinants affect on regular medication adherence in HD patients; some of these determinants include knowledge about diet therapy, socioeconomic status, health beliefs, attitudes toward treatment, and culture [14]. Treatment non-adherence compromises treatment effects, increases morbidity, mortality, hospitalization, and impose a significant economic burden on the health care system and indicated the necessary of the implementation of medication adherence promotion programs [15].

Basic behavior change interventions can be effective in clinical medicine and public health; meanwhile, identifying the effective determinants of a health behavior is very important in the development, implementation and evaluation of evidence-based health interventions [16]. The behavior change model, in order to treatment adherence, should be able to effectively plan for behavior change based on capability, environmental and social opportunities, as well as motivational conditions. To that end, The COM-B model introduces behavior as part of an interacting system including capability, opportunity, and motivation. Capability is defined as a person’s physical (physical strength, skill or stamina) and psychological (having the knowledge and skills to perform the behavior, and the capacity to perform essential thought processes such as understanding and reasoning) capacity to perform behavior. Motivation refers to internal processes that affect decision-making and behavior, and its two main components include reflective motivation (reflective process involved in planning) and automatic motivation (automatic processes such as impulses and inhibition). Opportunity is defined as all external factors that either improve behavior or make it possible to occur. Opportunity includes physical opportunity created in the environment (i.e. time, financial resources, access and signs) and social opportunity resulting from the cultural environment [17, 18]. COM-B model has been used to understand the influencing factors on behavior and also to develop interventions in treatment adherence behaviors among kidney patients [19–22].

Despite the evidence that treatment adherence can increase the effectiveness of HD and lead to improved clinical outcomes [5], however studies indicated a high rate of treatment non-adherence among HD patients [13]. Considering the lack of evidence-based studies in Iran, the aim of this study was to identify the determinants influencing medication adherence among ESRD patients in western Iran based on the COM-B model.

## Materials and methods

### Participants and study design

This research was conducted in the city of Kermanshah in the west of Iran, during 2021. This study adopted a cross-sectional design that guided by the COM-B framework. The study was conducted during the covid-19 pandemic. The data was collected in compliance with the covid-19 prevention guidelines. The study consisted of two steps.

In the first step, COM-B components of patients undergoing HD therapy were extracted through literature review (database search). This step was the basis for the development of quantitative tools to measure the components of COM-B. Our assessment of medication adherence among CKD patients was performed using the Behavior Change Wheel (BCW) and the Theoretical Domains Framework (TDF). A key advantage of using BCW is that it encourages program designers to comprehensively consider all options for intervention and then systematically select those that are best for intervention [23]. Also, TDF can be used to identify determinants affecting a behavior, while COM-B facilitates mapping of these determinants corresponding interventions. The TDF can be directly transposed to the COM-B Behavior Change Wheel, thereby eliminating much of the “guesswork” needed to determine strategies and/or implementation methods [24]. In the current study, effective determinants on medication adherence behaviors among CKD patients based on the literature review toward the COM-B model in the development of interventions among CKD patients [19–22] and also, literature review regarding the determinants affecting treatment adherence behaviors among CKD patients [25–30] was extracted. English articles published during 2017 to 2021 on the first five pages of Google Scholar, PubMed and Scopus databases were reviewed with search terms (“Hemodialysis” AND “Adherence”) (“Cognitive Determinants”) AND (“Capability-Opportunity-Motivation and Behavior Model” OR “COM-B”). Articles in non-English language, review reports, lack of access to full text, and duplicate sources were considered as exclusion criteria. After the end of the search, the titles of the sources were checked, and duplicate sources and unrelated items were removed from the research. Then the abstract of the remaining sources was carefully studied. Finally, full text references were reviewed. Then, according to their role in each of the components of the COM-B model, they were categorized. This process enabled us to gain insight into what needs to change for medication adherence behaviors to occur among CKD patients. It was also the basis for the development of quantitative instruments to measure the components of COM-B.

In the second step, a quantitative measurement tool was developed and its validity and reliability were confirmed. Then, using the validated tool, a cross-sectional study was conducted among 260 kidney patients undergoing HD therapy.

Inclusion criteria include: diagnosis of patients in ESRD, HD therapy for at least six months, receiving HD for three to four hours per session and three times a week, ability to answer the questionnaire items, willingness and informed consent to participate in the study. Patients on transient HD due to acute renal failure were excluded. The participants were selected by convenience sampling from patients referred to the dialysis unit of Imam Reza Hospital in Kermanshah, the west of Iran, during 2021. Out of 260 selected patients, 229 patients consciously completed the questionnaire and the response rate was 88%. The participants were informed about the confidentiality of the information and the purpose of the project and willingly enrolled in the study and signed a written consent form. The Ethics Committee of Kermanshah University of Medical Sciences approved this study.

### Measurements

The tool used in this research was a written questionnaire and data was collected based on an interview. Structured interviews were conducted by the fourth author using a scale which psychometric was confirmed by validity and reliability tests. Participants filled out the questionnaire before the HD.

The questionnaire had two parts, the first part related to demographic characteristics including; age, gender, marital status, education, job, income, health insurance, medication duration and dialysis duration. The second part of the questionnaire was based on the determinants related to the components of the COM-B model. The tool was developed using TDF with domains mapped onto COM-B components [24]. For this purpose, as previously explained, a literature review was used in relation to the determinants of treatment adherence among CKD patients. Finally, 21 items were extracted. Thus, determinants related to the capability component include knowledge (4 items; e.g., I have enough information about taking my medications regularly and on time), self-efficacy (2 items; e.g., I believe I can overcome my impatience because of long time medication), attitude (2 items; e.g., I do not believe that prescription medication are effective), perceived barrier (2 items; e.g., Because of being very busy during the day, use of my medication on schedule every day is difficult for me), and perceived risk (2 items; e.g., I believe the side effects of HD will be severe for me if I do not take my medications regularly), totally, capability component was measured with 12 items and an alpha coefficient of 0.70. Social support as a determinant related to the opportunity component with 4 items (e.g., I believe that the emotional support of my friends regarding the use of my medication encourages me to have a better management in the regular use of my medication) and the alpha coefficient of 0.78 was measured. The motivation component was also measured with 1 item (I take my medicines regularly and on time so that I don’t have a life full of pain and discomfort). Medication adherence was also measured by 4 items (for example: I take my medication at the appointed time) and Cronbach’s alpha coefficient was 0.72. A five-point Likert-type scale was used for respondents ranging from 1 (strongly disagree) to 5 (strongly agree).

In the present study, face validity, content validity and internal consistency were evaluated. The face validity of the questionnaire was evaluated qualitatively. In this way, face-to-face interviews were conducted with 12 experts and the items were modified according to their comments. The components of the COM-B model questionnaire content validity was measured by both quantitative and qualitative methods. For this purpose, 12 experts were interviewed and their comments about the difficulty, relevance, and the ambiguity were examined, and the items modified based on their comments. As well as, in order to measure quantitative content validity, 12 other experts were interviewed to assess whether each item was “essential”, “useful but not essential”, or “not essential”. Based on the Lawshe table, the minimum value for acceptable content validity index (CVI) and content validity ratio (CVR) was considered 0.79 and 0.62, respectively [31]. The expert panel included two health policy makers, three nursing experts related to kidney patients, one health care expert, one general practitioner, one nephrology specialist and four experts in health education and health promotion. The internal consistency was measured by using Cronbach’s Coefficient Alpha of the various components of the COM-B model. Prior to conducting the main project, a pilot study was conducted to assess the utility of the instrumentation. The pilot study participants were 20 ESRD patients, similar to those who participated in the main study. The pilot study was conducted to obtain feedback about the clarity, length, comprehensiveness, and completion time of the various instruments, as well as collecting data to estimate the internal consistency of the measures.

### Data Analysis

SPSS version 16 statistical software was used for data analysis. Descriptive information was expressed with frequency, percentage, mean and Standard Deviation (SD). Independent Samples t Test, One-way analysis of variance and Pearson correlation coefficient were used to measure the relationship between medication adherence and demographic characteristics. Pearson correlation coefficient was also used to measure the correlation between the components of the COM-B model. The most important assumptions such as linearity and independency for quantitative outcome were assess and confirmed. Multiple linear regression was used to determine the predictors of the medication adherence (model 1). Variables that p value more than 0.25 were excluded to adjusted model and variables that p value lower than 0.25 were remain (model 2). Cronbach’s coefficient alpha was used to measure reliability.

## Results

The mean age of respondents was 50.52 years [95% CI: 48.71, 52.33], ranged from 20 to 75 years. More details of demographic characteristics of the participants are shown in Table 1. As well as, Table 1 shows the relationship between demographic characteristics and medication adherence. As can be seen in Table 1, the mean score of medication adherence is higher among patients with higher education (P = 0.009) and those who were employed (P < 0.001).

Post-hoc analysis was performed with the Least Significant Difference (LSD). Findings indicated that the mean medication adherence score of diploma patients was significantly higher than patients with primary school education (P = 0.004). Also, the patients with secondary school education had a significantly higher score than the primary school patients (P = 0.040). But the difference between patients with secondary school education and diploma was not significant (P = 0.649).

**Table 1:** Distribution of the demographic characteristics and relationship between demographic characteristics with medication adherence

Demographic characteristics		Number (%)	Mean	SD	f/t	P
Gender	Female	98 (42.8%)	11.88	2.29	-0.396	0.693
	Male	131 (57.2%)	12.00	2.32		
Marital Status	Single	66 (28.8%)	12.01	1.84	0.254	0.800
	Married	163 (71.2%)	11.92	2.48		
Education level	Primary school	106 (46.3%)	11.45	2.00	4.801	0.009
	Secondary school	50 (21.8%)	12.27	2.05		
	Diploma	73 (31.9%)	12.47	2.72		
Job	Jobless or Housewife	163 (71.2%)	11.56	2.33	-4.058	< 0.001
	Employed	66 (28.8%)	12.90	1.94		
Health insurance	No	26 (11.4%)	11.47	1.95	-1.046	0.297
	Yes	203 (88.6%)	12.01	2.34		

Table 2 shows the correlations between medication adherence with age, income, medication and dialysis duration. Significance levels at the 0.01 and 0.05 were the criteria for the analysis. Our results indicated medication adherence was significantly related to income (r = 0.176), while it was inversely and significantly related to the medication duration (r=-0.250).

**Table 2:** Correlation of medication adherence behavior with age, income, medication and dialysis duration

		Age	Income	Medication duration	Dialysis duration
Age	r	1			
Income	r	0.228^**^	1		
	P	0.001			
Medication duration	r	0.115	-0.021	1	
	P	0.084	0.760		
Dialysis duration	r	0.011	-0.113	0.498^**^	1
	P	0.872	0.098	< 0.001	
Medication adherence	r	-0.107	0.176^*^	-0.250^**^	-0.061
	P	0.116	0.010	< 0.001	0.375

*. Correlation is significant at the 0.05 level (2-tailed).

**. Correlation is significant at the 0.01 level (2-tailed).

The mean score of medication adherence was 11.95 [95% CI: 11.64, 12.26], ranged from 4 to 20; that shows the patients obtained 59.7% of the maximum obtainable score for the medication adherence.

Table 3 shows the details of correlation, mean and range of COM-B component scores. All components had a positive and significant correlation with medication adherence at a significance level of 1%.

**Table 3:** Correlation, mean, and SD of COM-B dimension

		Capability	Opportunity	Motivation	Mean (SD)	Range
Capability	r	1			39.51 (5.76)	12–60
Opportunity	r	0.402^**^	1		15.78 (2.50)	4–20
	P	< 0.001				
Motivation	r	0.403^**^	0.433^**^	1	3.83 (0.86)	1–5
	P	< 0.001	< 0.001			
Behavior (medication adherence)	r	0.436^**^	0.283^**^	0.381^**^	11.95 (2.30)	4–20
	P	< 0.001	< 0.001	< 0.001		

**. Correlation is significant at the 0.01 level (2-tailed).

The predictors of the medication adherence are shown in Table 4. Initially, Crude analysis was performed and non-significant variables (gender, marital status, health insurance, and dialysis duration) were removed from the model. The results of adjusted analysis are also presented in Table 4. As can see in Table 4, the motivation (Beta = 0.296), self-efficacy (Beta = 0.244) and knowledge (Beta = 0.157) had significant effects on medication adherence among the HD patients in Iran.

**Table 4:** Predictors of the medication adherence

	Model 1 (Crude)				Model 2 (Adjusted)			
	B	Std. Error	Beta	P	B	Std. Error	Beta	P
Age	-0.017	0.011	-0.107	0.116	0.001	0.011	0.010	0.894
Gender	0.125	0.316	0.027	0.693	-	-	-	-
Marital Status	-0.087	0.342	-0.017	0.800	-	-	-	-
Education Level	0.523	0.175	0.199	0.003	0.050	0.192	0.020	0.793
Job	1.345	0.331	0.266	< 0.001	0.478	0.347	0.101	0.170
Health insurance	0.532	0.509	0.071	0.297	-	-	-	-
Income	1.697	0.000	0.176	0.010	3.649	0.000	0.038	0.636
Medication duration	-0.076	0.020	-0.250	< 0.001	-0.027	0.020	-0.080	0.187
Dialysis duration	-0.029	0.032	-0.061	0.375	-	-	-	-
Capability (Knowledge)	0.217	0.044	0.330	< 0.001	0.102	0.049	0.157	0.039
Capability (Self-efficacy)	0.480	0.072	0.410	< 0.001	0.277	0.084	0.244	0.001
Capability (Attitude)	0.162	0.076	0.143	0.034	0.079	0.085	0.071	0.357
Capability (Perceived barrier)	-0.294	0.095	-0.209	0.002	-0.016	0.100	-0.011	0.877
Capability (Perceived risk)	0.339	0.077	0.287	< 0.001	0.105	0.088	0.093	0.235
Opportunity (Social support)	0.256	0.060	0.283	< 0.001	0.001	0.069	0.001	0.985
Motivation	1.017	0.167	0.381	< 0.001	0.751	0.218	0.296	0.001

## Discussion

Our findings indicated that patients obtained 59.7% of the maximum obtainable score for the medication adherence. Other studies reported treatment adherence rates of less than 50% [13]. The need to identify determinants influencing treatment adherence in order to develop, implement and evaluation of treatment adherence promotion programs among HD patients is clear. According to our knowledge, the present study is the first study in Iran that uses Michie’s BCW to identify the determinants affecting medication adherence among HD patients. The present study showed that motivation was the strongest predictor of medication adherence. In line with our findings, several studies pointed out the correlation and positive effect of motivation on the adherence to medication in patients undergoing HD therapy [24, 32]. Strengthening motivation, by empowering patients to play a more active role in self-care, can lead to the promotion of healthy behaviors and treatment adherence [33]. In this regard, Ok and Kutlu showed in their Randomized Controlled Trial (RCT) indicated that interventions in the field of strengthening motivation can play an important role in improving treatment adherence of HD patients [34]. Also, Mersha et al., in their study based on the COM-B model, identified automatic motivation as one of the most important factors of treatment adherence [35]. In the focus group study conducted by Cardol, has been mentioned the positive role of intrinsic motivation on the engagement of CKD patients in a healthy lifestyle [36]. Our findings confirm the usefulness of using Behavioral Change Methods (BCMs) or Behavior Change Techniques (BCTs) that are suitable for motivation in the development of interventions to promote treatment adherence among HD patients. Some BCMs for increasing motivation presented by Kok et al. [16] and Michie et al. [37] include self-reevaluation (encouraging the combination of both emotional and cognitive evaluation of the patient’s self-concept with and without healthy behavior), environmental reevaluation (encouraging to recognize the negative impact of unhealthy behavior and the positive impact of healthy behavior), and anticipating regret (stimulating people to focus on their feelings after unhealthy behavior). This is a useful finding that can be considered in the development of interventions to promote medication adherence among HD patients in Iran.

Moreover, our findings showed that the capability component (self-efficacy and knowledge determinants) was the second most effective predictor of medication adherence among HD patients. In line with the present finding, several studies have emphasized the role of the determinants of the component of capability on performing treatment adherence [28–30]. For example, Náfrádi et al. in their systematic review showed that high levels of self-efficacy consistently increase medication adherence in HD patients [28]. Sousa et al., in their study indicates understanding the benefits of treatment and knowledge are among the determinants of hemodialysis attendance [29]. In addition, Spies et al. also emphasized the importance of increasing knowledge among hemodialysis patients [30]. Therefore, in order to increase the level of medication adherence, health education professionals should provide patients with detailed explanations about the benefits of treatment adherence and strategies to overcome barrier to treatment adherence, while using self-efficacy strategies or methods. This can reduce the uncertainty of patients and ultimately increase the level of treatment adherence. Some BCMs that can be useful in this context include self-monitoring of behavior (encouraging the patient to maintain specified behaviors), goal setting (encouraging planning for what one will do), planning coping responses (making a list of possible obstacles and ways to overcome them), and discussion (encouraging to examine the issue in an open informal debate) [16] that can be used in the development of interventions.

Consistent with our findings, Kim and Kim in their study among 152 HD patients in Korea reported that higher education was associated with self-care compliance [38]. In another study among 100 HD patients, Kim and Cho showed the relationship between high level of education and greater adherence to treatment [39]. As well as, some studies reported that higher education can lead to an increase in self-efficacy and Perceived Behavior Control (PBC) [3]. Thus, it is recommended to develop and implement interventions, especially for patients with lower education, in order to improve the level of medication adherence.

Another of our findings was the lack of significant difference in medication adherence between men and women. Although in some studies it has been reported that women generally adhere better to treatment compared to men [3, 40]. It seems that the development of interventions to promote treatment adherence in Iran should be considered in both sex.

The amount of income was another factor that had a significant positive correlation with medication adherence. This finding is consistent with other studies that showed the positive effect of higher socioeconomic status on treatment adherence among HD patients [41, 42]. The development and implementation of interventions, especially in the socially and economically disadvantaged group, can be a strategy to reduce inequality in this field.

An interesting finding in the present study was the inverse significant correlation between medication adherence and medication duration. Also, medication adherence had an inverse correlation with increasing age. In contrast to our findings, Zaidi et al. showed in their two studies that with increasing dialysis duration and longer treatment, medication adherence increases [43, 44]. Ishaq et al. in their study also showed a positive relationship between the medication duration and treatment adherence [45]. As well as, Alhomayani et al. in their study in Saudi Arabia showed a significant correlation between the medication duration and treatment adherence in HD patients [46]. According to the findings of the mentioned studies, it seems that with the passage of time and seeing the benefits of treatment adherence, patients are naturally affected by the effects of treatment and it seems that this issue contributes to the increase in medication adherence. However, our findings were not in line with these studies. Our finding can be warning to health policy makers in Iran; and should be the focus of special attention.

### Strengths and limitations

For the first time in Iran, we used Michie’s BCW to identify the factors influencing drug adherence behavior among ESRD patients. However, our study also had limitations. First, we use the writing questionnaire to collect data, which can be associated with a percentage of error in the responses of the participants. Second, the present study was conducted only in a group of ESRD patients in western Iran and may not be generalizable to other ESRD patients. Third, we did not examine the mean of medications intake per day by ESRD patients. Finally, the current study was cross-sectional, one should be careful when interpreting the results because it does not investigate causality.

## Conclusion

The present study contributes to the development of the psychological understanding of the health-seeking behavior of ESRD patients in medication adherence. Our findings indicated the effective role of motivation, self-efficacy, and knowledge in predicting medication adherence among Iranian ESRD patients. COM-B model can be proposed as an integrated framework in predicting medication adherence among ESRD patients. Our findings provide theory-based recommendations that can help future clinical and research decision-making for the development, implementation, and evaluation of treatment adherence interventions in Iranian ESRD patients. The use of COM-B model can provide a comprehensive explanation about medication adherence in ESRD patients. Future research should be focus on increasing motivation, self-efficacy and knowledge of Iranian ESRD patients in order to increasing medication adherence.

## Data Availability

The datasets used and/or analyzed during the current study are available from the corresponding author on reasonable request.

## List of Abbreviation

BCMs: Behavioral Change Methods
BCTs: Behavior Change Techniques
BCW: Behavior Change Wheel
CKD: Chronic Kidney Disease
COM-B: Capability-Opportunity-Motivation and Behavior
ESRD: End Stage Renal Disease
HD: Hemodialysis
LSD: Least Significant Difference
PBC: Perceived Behavior Control
SD: Standard Deviation
TDF: Theoretical Domains Framework
WHO: World Health Organization
